# Significance of Epicardial and Intrathoracic Adipose Tissue Volume among Type 1 Diabetes Patients in the DCCT/EDIC: A Pilot Study

**DOI:** 10.1371/journal.pone.0159958

**Published:** 2016-07-26

**Authors:** Sirous Darabian, Jye-Yu C. Backlund, Patricia A. Cleary, Nasim Sheidaee, Ionut Bebu, John M. Lachin, Matthew J. Budoff, DCCT/EDIC Research Group

**Affiliations:** 1 Los Angeles Biomedical Research institute, Torrance, California, United States of America; 2 The Biostatistics Center, the George Washington University, Rockville, Maryland, United States of America; Cedars Sinai Medical Center, UNITED STATES

## Abstract

**Introduction:**

Type 1 diabetes (T1DM) patients are at increased risk of coronary artery disease (CAD). This pilot study sought to evaluate the relationship between epicardial adipose tissue (EAT) and intra-thoracic adipose tissue (IAT) volumes and cardio-metabolic risk factors in T1DM.

**Method:**

EAT/IAT volumes in 100 patients, underwent non-contrast cardiac computed tomography in the Diabetes Control and Complications Trial /Epidemiology of Diabetes Interventions and Complications (DCCT/EDIC) study were measured by a certified reader. Fat was defined as pixels’ density of -30 to -190 Hounsfield Unit. The associations were assessed using–Pearson partial correlation and linear regression models adjusted for gender and age with inverse probability sample weighting.

**Results:**

The weighted mean age was 43 years (range 32–57) and 53% were male. Adjusted for gender, Pearson correlation analysis showed a significant correlation between age and EAT/IAT volumes (both p<0.001). After adjusting for gender and age, participants with greater BMI, higher waist to hip ratio (WTH), higher weighted HbA1c, elevated triglyceride level, and a history of albumin excretion rate of equal or greater than 300 mg/d (AER≥300) or end stage renal disease (ESRD) had significantly larger EAT/IAT volumes.

**Conclusion:**

T1DM patients with greater BMI, WTH ratio, weighted HbA1c level, triglyceride level and AER≥300/ESRD had significantly larger EAT/IAT volumes. Larger sample size studies are recommended to evaluate independency.

## Introduction

Several large studies have shown a high prevalence of cardiovascular disease (CVD) among participants with type 1 diabetes (T1DM). [1–3]While the incident of CVD in 35–44 years old age non-diabetic population has been reported as 0.1%, the Pittsburgh Epidemiology of Diabetes Complications (EDC) study reported 0.98% per year incidence of major coronary artery disease (CAD) events, among 28–38 years with T1DM.[4] Moreover, a minimum 10-fold increase in mortality due to cardiovascular disease among T1DM patients was reported.[5] Furthermore, there is an independent association between CAD events and major coronary artery disease risk factor including lipid profile, hypertension, smoking, and nephropathy and markers of inflammation among patients with T1DM. [4,6–8]

Adipose tissue, as the biggest endocrine organ in the body, is involved with the production of many bioactive molecules. Visceral adipose tissue and its adipose-tissue resident macrophages produce pro-inflammatory cytokines like tumor necrosis factor-alpha (TNF-alpha) and interleukin-6 (IL-6). These adipose tissue inflammation mediators may promote endothelial dysfunction as well as development and progression of atherosclerosis. Nevertheless, the role of other adipose tissue depot, such as epicardial adipose tissue (EAT) and intra-thoracic adipose tissue (IAT), play in the development of CAD has not been established. This pilot study sought to examine the relation between EAT and IAT volumes with selected cardio-metabolic risk factors, treatment method, and glycemic levels among T1DM patients.

## Study Design and Method

### Ethics statement

All human research was approved by the relevant institutional review boards, and conducted according to the Declaration of Helsinki. Institutional Review Board (IRB) approval was obtained by each of the individual clinical centers (Albert Einstein College of Medicine, Case Western Reserve University, Weill Cornell Medical College, Henry Ford Health System, International Diabetes Center, Joslin Diabetes Center, Massachusetts General Hospital, Mayo Foundation, Medical University of South Carolina, Northwestern University, University of California, San Diego, University of Iowa, University of Maryland School of Medicine, University of Michigan, University of Minnesota, University of Missouri, University of Pennsylvania, University of Pittsburgh, University of South Florida, University of Tennessee, University of Texas Southwestern Medical Center at Dallas, University of Toronto, University of Washington, University of Western Ontario, Vanderbilt University, Washington University, St. Louis, Yale University School of Medicine), Computed Tomography Reading Center (Harbor UCLA Research and Education Institute), and the Data Coordinating Center (the George Washington University Biostatistics Center). Written informed consent was provided by each of the study participants at the clinical centers. Consent for minor participants at study screening and randomization was provided by the minor’s parent or legal guardian, as directed by local institutional guidelines.

### Study population

This pilot study employed 100 selected participants who participated in the computed tomography (CT) protocol in the Diabetes Control and Complications Trial /Epidemiology of Diabetes Interventions and Complications (DCCT/EDIC) study. The details of the CT protocol have been previously described [9]. The 100 patients were obtained from a case-control sample of subjects who participated in a cardiac magnetic resonance imaging (cMRI) study and we had access on their non-contrast cardiac CT images. The minimal dataset containing EAT and IAT and selected covariates was available in the Supporting Information file “S1 File”.

The EDIC study[10] began in 1994 and is an ongoing, prospective observational follow-up of the DCCT cohort[11], which was a controlled clinical trial of intensive versus conventional diabetes treatment in 1441 subjects with type 1 diabetes. Based on the treatment protocols, patients were randomized into two groups of intensive and conventional treatment groups. The intensive treatment was designed to maintain glucose levels as close to the normal range as possible utilizing three or more daily insulin injections or an insulin infusion pump, with dose adjustments guided by frequent self-monitoring of blood glucose. Conventional treatment consisted of 1–2 daily insulin injections with no specific target level[12].

### Assessment of Covariates

During DCCT, participants had an annual medical history and physical examination, and laboratory testing for fasting lipid levels, urinary albumin excretion rate (AER) and other risk factors for cardiovascular disease[11]. Glycated hemoglobin values (HbA1c) were measured quarterly during DCCT[13]. During EDIC, annual medical histories were obtained, physical exams performed and HbA1c levels were measured. Lipid profiles and urinary AER were measured in alternate years [10]^.^ Due to the differences in the intervals between visits during DCCT and EDIC, weighted mean HbA1c was computed with weights proportional to the time interval between values.

### Measurements of Adipose Tissue

**Fig 1:**
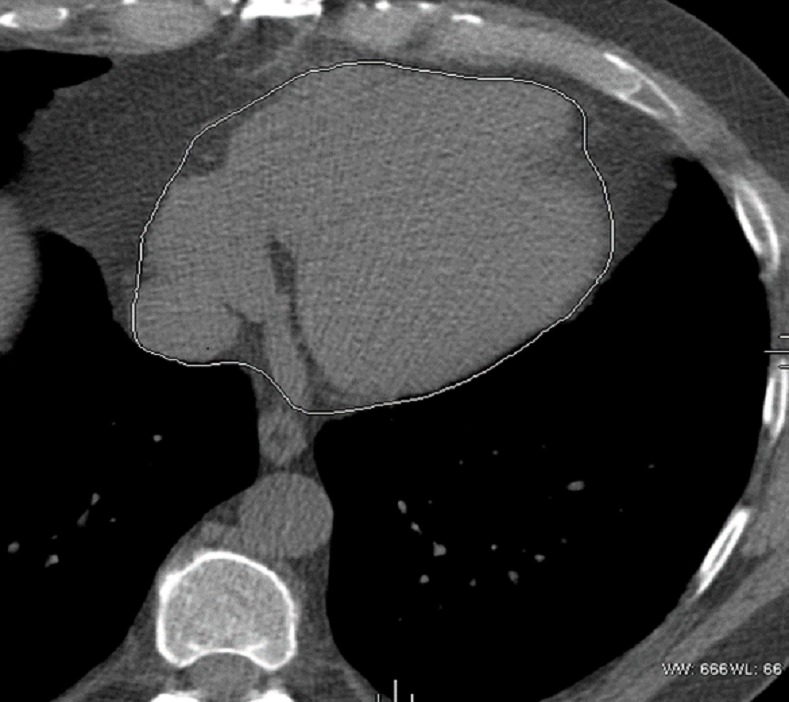
Pericardial Tracing to Define Epicardial Fat Volume.

All adipose tissue measurements were performed on the axial slices of non-contrast cardiac CT using GE Advantage Windows 4.6 Workstations “Fig 1“. EAT was defined as the adipose tissue between the surface of the heart and the visceral pericardium. EAT measurements were performed on axial images starting from 10mm above the superior extent of the left main ostium to the lower most slice containing a part of pericardial sac. The reader defined the boundary of the EAT by manually tracing the pericardium. IAT measurement was performed utilizing the same superior boundary as described above. The diaphragm defined the inferior boundary for IAT. The anterior border of the volume was defined by the chest wall and posterior border by the aorta, bronchi and esophagus. Adipose tissue present in the posterior mediastinum and para-aortic adipose tissue was not included in the IAT measurements. The observer had interactive access to the coronal and sagittal images as an assist for accurate measurements. Adipose tissue was discerned from the remainders with a threshold of -190 to -30 Hounsfield units. This method provides a high repeatability, and reproducibility rate.[14] One well-trained and certified cardiac CT reader, blinded to the patient characteristics, and other data, did all adipose tissue measurements.

### Statistical Analysis

The outcomes of this study are epicardial and intra thoracic adipose tissue volumes (EAT and IAT). Clinical characteristics (predictors) were evaluated from the last available visit prior to or concurrent with the cardiac CT. The predictors are attained age, gender, attained T1DM duration, BMI, waist to hip ratio, total daily insulin dose, systolic and diastolic blood pressures, triglyceride level, high density lipoprotein (HDL) level, estimated glomerular filtration rate (eGFR), macroalbuminuria (albumin excretion rate (AER) equal or greater than 300 or end stage renal disease (ESRD) at any time, and weighted mean HbA1c.

All analyses were weighted using the inverse of the probability of selection in the sample of 100 out of the full cohort of 1205 subjects with CT scans during EDIC so as to yield unbiased estimates. [14, 15] The sampling probabilities were calculated for each subject in the pilot study based on gender and tertiles of age, six strata total. The Pearson correlation coefficients adjusting for gender were performed to assess the correlation between EAT/IAT and individual risk factors. In addition, the association of each covariate separately was assessed using a minimally adjusted model (adjusted for age and gender). After the inverse probability sample weight adjustment, the sample cohort of 100 shared the similar summary statistics for clinical characteristics with the full cohort (53% males, mean age 43 years, and mean HbA1c 8.1%) “Table 1”.

**Table 1:** Similar Clinical Characteristics for Weighted Sample and the Full Cohort.

	Weighted Sample*		Full Cohort	
Variable^#^	Mean	S.D.	Mean	S.D.
N	100		1205	
Female (%)	47.5		47.5	
Attained Age (years)	43	6	43	7
Intensive Treatment (%)	55.2		49.5	
Insulin Dose (Units/kg/day) ^‡^	0.67	0.27	0.68	0.23
Attained Duration of IDDM (years)^†^	22.2	4.9	21.0	4.9
Mean Glycosylated hemoglobin during DCCT/EDIC (%)	8.1	0.9	8.1	1.1
Body Mass Index (kg/m^2^)^‡^	27.2	4.2	27.5	4.4
Waist/Hip Ratio^‡^	0.85	0.07	0.85	0.09
HDL Cholesterol (mg/dl)^‡^	54	12	56	15
LDL Cholesterol (mg/dl) ^‡^	110	27	111	29
Triglyceride (mg/dl)^‡^	80	40	90	62
AER≥300 or ESRD (%)^‡^	9.2		8.5	
eGFR^‡^	106	14	103	16
**Anti-hypertensive (yes)(%)**	30.7		33.6	
**Lipid lowering medication (yes)(%)**	30.1		22.2	
Epicardial Fat Volume (mm^3^)	38.5	16.6	N/A	N/A
Intrathoracic Fat Volume (mm^3^)	50.8	25.3	N/A	N/A

^#^ At time of CT scan in 60 (60%) of subjects or at a prior visit in 40(40%) conducted within 20 months (average 4 months) before or after the CT scan.

N/A Not Applicable

* Adjusting for the inverse probability sample weight.

^†^ At time of CT scan or prior visit.

^‡^ Measured at the last available visit prior to or at cardiac CT.

All analyses were performed using SAS software (version 9.2; SAS Institute, Cary, NC). P values <0.05 were considered statistically significant.

## Results

**Fig 2:**
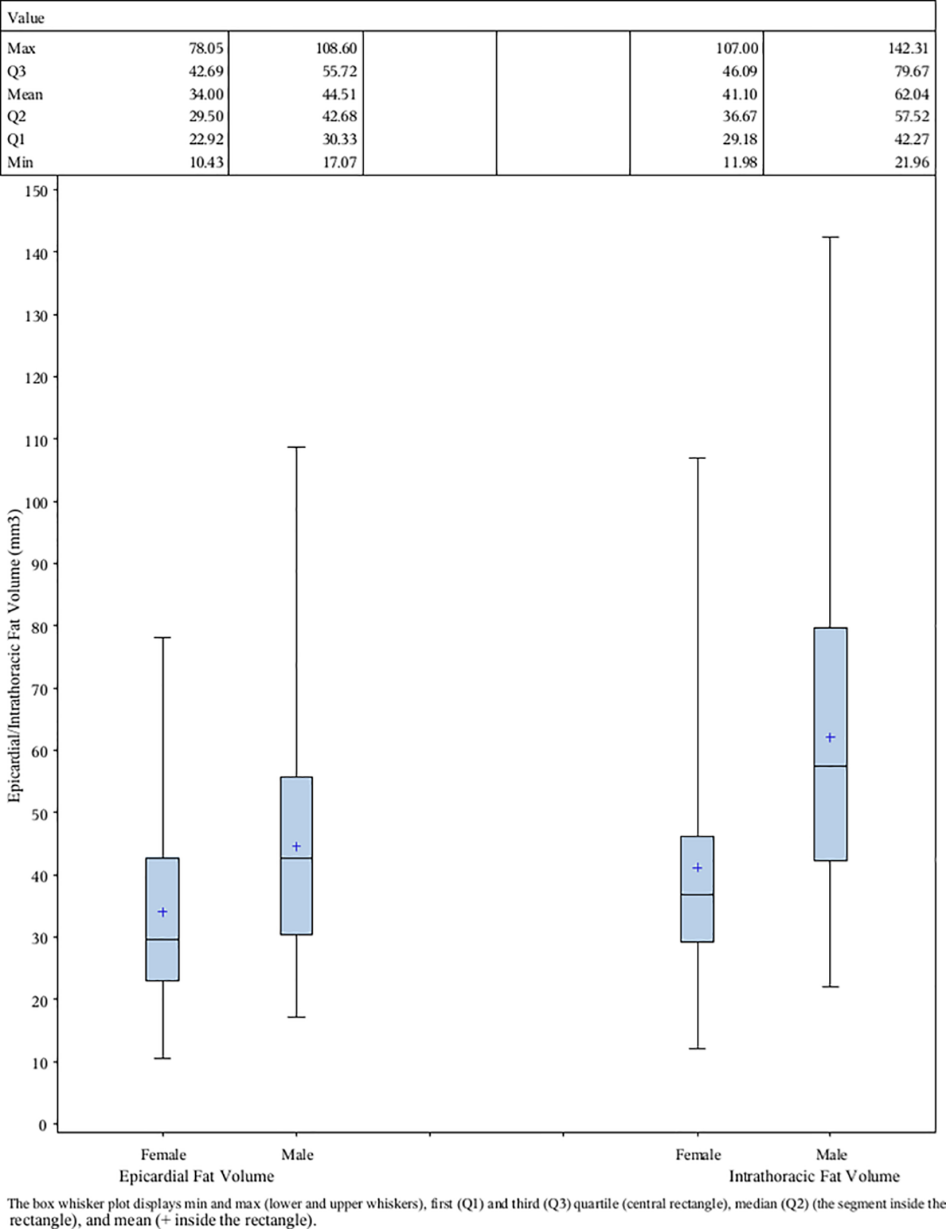
Epicardial/Intrathoracic Fat Volume with Sample Weight Adjustment by Gender. The box whisker plot displays min and max (lower and upper whiskers), first (Q1) and third (Q3) quartile (central rectangle), median (the segment inside the rectangle), and mean (○/+ inside the rectangle).

Of the 100 patients, weighted mean aged 43 years (range 32–57), 53% were male “Table 1“. The mean EAT and IAT volumes were significantly different in women and men; 34±15 and 41±19 in women and 43±20 and 60±33 mm3 in men, respectively (p = 0.016 and p = 0.0003) “Fig 2“. Furthermore, EAT and IAT volumes were shown to correlate with age (r = 0.35 and 0.27, respectively; p = 0.0018 and 0.014). No significant differences in volumes of EAT and IAT were noted based on original DCCT treatment groups, smoking, insulin dose or duration of T1DM. Weighted mean HbA1c during DCCT/EDIC had a significant positive correlation with both EAT and IAT volumes after adjusting for gender. After adjusting for age and gender, the analyses showed significant associations between EAT and IAT volumes and BMI, waist to hip ratio, triglycerides, weighted mean HbA1c, and AER≥300/ ESRD (ever) “Table 2“. No association was noted between EAT and IAT and HDL cholesterol, smoking, systolic, or diastolic blood pressures. No any interaction between each covariate and sex was significant in epicardial fat volume. Only one interaction between AER> = 300/ESRD and sex reached the significance level (p = 0.0093) in intrathoracic fat volume. Males patients who had AER > = 300/ESRD had the highest intrathoracic fat volume (88 mm^3^; N = 6) while females patients who had AER > = 300/ESRD had the lowest intrathoracic fat volume(36 mm^3^; N = 2). The most significant risk factors, waist, weighted mean HbA1c and triglycerides were adjusted in the multivariate model besides age and gender. Weighted mean HbA1c and triglycerides were no longer significant in the multivariate model while the waist remained significant (data not shown). In addition, while 31% of 1205 patients in full cohort had no coronary artery calcium deposit, 28% of our sample population showed zero coronary artery calcium score. After adjusting for gender and sample weight, both partial correlation coefficient and p-value between coronary artery calcium and EAT and IAT volumes was the same, 0.18 and 0.08, respectively. In addition, majority of subjects with type 1 diabetes in the full cohort were northern European ancestry (i.e. Caucasian), characteristics of type 1 diabetes. The fraction of other races in full cohort and sample were 3.3% and 3.0%, respectively.

**Table 2:** Pearson Correlation* and Minimally Adjusted Model^@^: Epicardial Fat Volume and Intrathoracic Fat Volume Associated with Selected Covariates.

Covariate prior to or at CT scan	Epicardial Fat Volume (mm^3^)				Intrathoracic Fat Volume (mm^3^)			
	Correlation		Model		Correlation		Model	
	coefficient	p-value	Estimate±SE	p-value	coefficient	p-value	Estimate±SE	p-value
Gender^†^ (males vs. females)	-		9.2 ± 3.7	0.0159	-		19.3 ± 5.2	0.0003
Age^‡^ (years)	0.35	0.0005	0.93 ± 0.29	0.0018	0.27	0.0069	1.1 ± 0.4	0.0140
Duration^‡^ (years)	-0.18	0.0804	-0.5 ± 0.4	0.1799	-0.18	0.0680	-0.8 ± 0.5	0.1344
Treatment(Conventional vs. Intensive)	N/A	N/A	-4.0 ± 3.3	0.2384	N/A	N/A	-4.6 ± 5.1	0.3696
Smoking (yes vs. no)	N/A	N/A	8.1 ± 4.9	0.1040	N/A	N/A	9.8 ± 7.0	0.1627
BMI (kg/m^2^)	0.39	< 0.0001	1.6 ± 0.4	<0.0001	0.40	<0.0001	2.4 ± 0.6	<0.0001
Waist (cm)	0.50	< 0.0001	0.80 ± 0.14	< 0.0001	0.50	< 0.0001	1.17 ± 0.20	< 0.0001
Waist/Hip Ratio^$^	0.58	< 0.0001	15.4 ± 2.4	< 0.0001	0.60	< 0.0001	23.8 ± 4.0	< 0.0001
Weighted HbA1C during DCCT/EDIC (%)	0.22	0.0271	4.4 ± 1.6	0.0075	0.18	0.0721	5.2 ± 2.2	0.0196
Insulin dose (units/kg/day)	0.04	0.6991	9.5 ± 5.7	0.0982	0.03	0.7847	10.6 ± 8.3	0.2075
Systolic blood pressure (mm Hg)	0.07	0.5057	0.11 ± 0.16	0.4803	0.07	0.4645	0.17 ± 0.23	0.4501
Lipid-HDL Cholesterol (mg/dl)	-0.21	0.0335	-0.33 ± 0.19	0.0795	-0.21	0.0403	-0.46 ± 0.28	0.1020
Lipid-LDL Cholesterol (mg/dl)	0.08	0.4358	-0.01 ± 0.07	0.8672	-0.07	0.4639	-0.02 ± 0.10	0.8125
Triglycerides (mg/dl)	0.21	0.0330	0.09 ± 0.04	0.0273	0.20	0.0475	0.13 ± 0.07	0.0579
eGFR	-0.23	0.0231	-0.08 ± 0.09	0.4113	-0.16	0.1117	-0.05 ± 0.13	0.7138
AER≥300 or ESRD (ever)	N/A	N/A	10.3 ± 5.1	0.0450	N/A	N/A	20.5 ± 9.5	0.0338
Anti-hypertensive (yes vs. no)	N/A	N/A	3.6 ± 3.6	0.3210	N/A	N/A	7.5 ± 5.4	0.1676
Lipid lowering medication (yes vs. no)	N/A	N/A	9.0 ± 4.0	0.0282	N/A	N/A	11.3 ± 6.2	0.0696

N/A Pearson correlation was Not Applicable for binary variables.

* Pearson partial correlation was adjusted for gender and corrected with sample weight.

^@^ All models were adjusted for gender and age and corrected with sample weight unless otherwise indicated. A separate model was computed for each covariate.

^†^ Model included gender only.

^‡^ Model was adjusted for gender.

^$^ Every 0.1 unit change of waist/hip ratio in the minimally adjusted model.

## Discussion

Not only has the volume of the fat, but also the body fat distribution associated with CAD. BMI and waist to hip ratio have been used frequently to assess the impact of the obesity on coronary artery disease. However, there are many studies pointing to the possibility that use of BMI may be responsible for the observed obesity paradox. Obesity paradox is a term indicating high BMI may be protective and associated with greater survival in certain populations.[15,16] A recent published results of a Canadian cohort study over 54420 aged 40 or above participants, suggested that BMI acts as a misleading factor. In this study, adults with higher BMI survived more than their counterpart. But, DXA-derived body fat percentage revealed participants with higher fat percentage had worse outcomes.[17] In contrast, visceral adipose tissue and more specifically EAT and IAT showed a stronger association with cardiovascular health and events. [18–20] While, based on the current guidelines, a non-contrast cardiac CT and evaluation of coronary artery calcium score is recommended for screening of CAD among diabetic patients, it is possible to take the advantage of having EAT and IAT volumes to assess the risk of the future events. A study in 998 apparently healthy individuals, who were randomly selected from 6814 Multi-Ethnic Study of Atherosclerosis (MESA) participants revealed that deposit of fat around the heart strongly predicts incident coronary artery disease, independent of the BMI and major CAD risk factors.[21] Noteworthy, in contrast to many previous studies, a multi-centric study was not able to find any association between epicardial fat and the presence and extent of CAC by CT, presence of obstructive CAD by cardiac catheterization, or myocardial perfusion abnormalities by SPECT. [22] However, different from most of the other studies and the standards that was defined earlier, a range of -45 to -195 HU was used to define the fat. Based on the detailed studies, current standards recommended -30 to -190 HU for fat measurement.[23,24] It means they did not considered any mass between -30 to -45 as fat. Since this usually includes a significant mass inside the pericardium, it is possible that the difference made the results very different from the other studies. We have considered a range of -30 to -190 HU for fat.

Previous studies showed significant differences between men and women in both the fat metabolism and pattern of fat distribution. Usually, women have a greater body fat proportion than men. Also, subcutaneous fat storage is greater in female than male, while visceral fat deposits are more prominent in men [25]. Our pilot study showed male gender had a strong association with greater volumes of EAT and IAT. Also, it showed among T1DM, independent of age, males have greater EAT and IAT volumes than females. The difference in the pattern of fat distribution will help to detect patients with greater risk of cardiovascular disease.

Obesity and greater body fat volume have been considered one of the primary adverse effects of insulin therapy in both Type 1 and 2 diabetes.[26,27] In the Diabetes Control and Complications Trial, whereas waist to hip ratios did not differ, T1DM patients receiving intensive insulin therapy significantly gained body weight compare to patients receiving conventional therapy. Our study shows no association between total daily insulin dose and EAT or IAT fat volumes. Likewise, neither the DCCT treatment assignment (intensive vs. conventional) nor the duration of T1DM showed a relation with EAT or IAT volumes. However, interestingly, weighted HbA1C during DCCT/EDIC showed to have a positive association with these visceral fat volumes.

### Limitations

We were not able to evaluate the independent association of EAT and IAT volumes with more CVD risk factors due to the small sample size.

## Conclusion

Among T1DM patients, accumulation of adipose tissue in epicardial and intra-thoracic spaces were highly associated with greater BMI, bigger waist to hip ratio, greater weighted HbA1c, elevated triglyceride level and a history of AER≥300 or ESRD). Male individuals with T1DM had significantly larger volumes of EAT and IAT. Further studies with larger size and considering hard events is advised.

## Supporting Information

S1 File Minimal data set with EAT and IAT and selected covariates. (PDF)
