# Supplementary material for: Significance of Epicardial and Intrathoracic Adipose Tissue Volume among Type 1 Diabetes Patients in the DCCT/EDIC: A Pilot Study
Source: PLoS One. 2016 Jul 26;11(7):e0159958. doi: 10.1371/journal.pone.0159958 (PMC4961378; doi:10.1371/journal.pone.0159958)
Supplement: S1 File — (PDF) [file pone.0159958.s001.pdf]

S1 File: Minimal dataset with EAT and IAT and selected covariates

Epicardial Fat Volume (EAT) (mm^3)	Intrathoracic Fat Volume (IAT) (mm^3)	Body mass index (kg/m^2)	Sex	Attained age (years)	Weighted mean HbA1c (%)
37.58	45.2	24.2865	M	48	8.0043
37.53	43.28	25.3352	M	46	7.0925
50.98	58.34	29.9603	M	47	9.5131
43.381	53.657	34.8756	F	32	10.2227
18.88	22.24	23.3664	M	44	8.8935
72.52	117.45	31.218	M	35	7.9215
27.48	36.89	30.9018	F	42	7.7037
40.08	43.28	24.331	M	34	9.2027
20.4	24.46	21.3799	F	35	8.0794
61.05	87.25	29.7806	M	52	7.8873
23.257	26.834	27.6995	M	32	7.1389
10.43	11.978	24.7557	F	37	7.2375
30.06	40.73	23.9146	M	46	6.841
59.24	80.67	27.1359	M	36	8.3372
27.75	39.23	25.0088	M	56	8.7795
46.95	54.04	26.285	M	57	8.2643
44.57	51.31	25.0568	M	50	8.0743
43.39	60.14	24.8075	M	43	6.9303
55.3	59.48	38.7523	F	43	8.0318
